# Assessing the knowledge, practices and collaborative readiness of community pharmacists’ management of progressive joint disorders in South Africa

**DOI:** 10.4102/hsag.v31i0.3211

**Published:** 2026-02-23

**Authors:** Tumelo Modau, Demitri Constantinou, Ané Orchard

**Affiliations:** 1 Department of Pharmacy and Pharmacology, Faculty of Health Sciences, University of the Witwatersrand, Johannesburg, South Africa; 2 Department of Exercise Science and Sports Medicine, Faculty of Health Sciences, University of the Witwatersrand, Johannesburg, South Africa

**Keywords:** allied healthcare providers, guidelines, multidisciplinary, musculoskeletal conditions, pharmacy

## Abstract

**Background:**

Progressive joint disorders (PJDs) are a leading cause of chronic pain and disability, requiring multidisciplinary management. Community pharmacists are well positioned to support patients through education, medication management and referrals, yet gaps in referral practices and interprofessional collaboration persist.

**Aim:**

To assess South African community pharmacists’ referral practices in managing PJDs and their knowledge of other healthcare providers’ roles, with the goal of identifying opportunities to enhance collaboration and inform the development of referral guidelines.

**Setting:**

Private community pharmacies across South Africa.

**Methods:**

A cross-sectional survey was conducted using a structured online questionnaire distributed nationally. Data on demographics, familiarity with PJDs, management practices, referral behaviours and barriers to collaboration were analysed using descriptive and inferential statistics.

**Results:**

Of 373 participants, 84.5% were familiar with PJDs, most commonly gout, osteoarthritis and rheumatoid arthritis. Over 60% did not use formal guidelines for PJD management. There was no significant association between guideline use and familiarity with other providers’ roles (*p* = 0.96). Referrals were predominantly to medical doctors with limited engagement with other health professionals. Barriers included a lack of referral protocols and limited knowledge of other providers’ roles. Most pharmacists expressed a willingness to improve care through better collaboration and education.

**Conclusion:**

Pharmacists play a pivotal role in PJD management and require better training, awareness and structured referral pathways to enhance multidisciplinary care and optimise patient outcomes.

**Contribution:**

This study highlights the need for evidence-based referral guidelines and interprofessional education in pharmacy curricula to strengthen pharmacists’ role in multidisciplinary PJD care.

## Introduction

Progressive joint disorders (PJDs) may be inflammatory and/or degenerative in nature and are among the leading causes of chronic musculoskeletal pain and disability globally (Alzahrani et al. 2022; Flynn 2020). They place a significant burden on healthcare systems, contribute to work absence and are increasingly prevalent because of ageing, obesity and physical inactivity (Alzahrani et al. 2022). In severe cases, PJDs impair daily functioning and may result in early retirement (Alzahrani et al. 2022; Flynn 2020). South Africa is no exception, with rising rates of non-communicable diseases compounded by an ageing population and the challenges of multi-morbidity (Coetzee, Giljam-Enright & Morris 2020).

The management of PJDs requires a multimodal approach, integrating pharmacologic and non-pharmacologic interventions consisting of physical therapy, cognitive-behavioural therapy, lifestyle modifications and, in severe cases, surgical interventions (Flynn 2020). Community pharmacists are well positioned to identify patients who could benefit from multimodal approaches, such as those with undiagnosed PJDs, unresolved pain or suboptimal outcomes from pharmacotherapy (Mishriky, Stupans & Chan 2019; Thapa et al. 2021). It is important that community pharmacists support the early identification and management of PJDs through patient education, medication advice and signposting for referrals (Simons et al. 2022). Many of these conditions often require early intervention to delay disease progression, reverse joint damage and minimise disability (Simons et al. 2022).

Community pharmacists have a pivotal role to play in the referral of these patients; however, studies indicate challenges in their engagement with other healthcare providers (HCPs) (Mishriky et al. 2019; Taylor et al. 2022). International evidence suggests that pharmacists often lack formal guidelines or systematic referral protocols, which hinders their ability to effectively collaborate with physiotherapists, occupational therapists, biokineticists (exercise physiologist), dieticians or other HCPs best suited to support integrated management of PJDs (Mishriky et al. 2019; Taylor et al. 2022). This situation is likely to mirror local realities, given the limited interprofessional infrastructure in South Africa (Coetzee et al. 2020). Furthermore, reports indicate that pharmacists are not well versed in the roles of these HCPs (Taylor et al. 2022; Takahashi et al. 2019). This lack of knowledge may be because of outdated, uni-professional undergraduate training models that neglect to integrate interprofessional education into the curriculum, resulting in graduates who are not collaborative ready (El-Awaisi et al. 2018).

Over the past few decades, community pharmacists in practice have relied on informal networks or personal judgement instead of informed referral practices when managing PJDs, and this highlights a critical area for improvement (Bosson et al. 2023; Cottrell et al. 2021). Training and resources to enhance pharmacists’ familiarity with referral processes and other HCPs’ roles could facilitate collaboration and appropriate referrals to these HCPs and significantly improve the management of PJDs (Cottrell et al. 2021; Kredo et al. 2020).

Research has, in fact, shown that clear, practical and evidence-based referral guidelines can bridge the gaps in the lack of referrals, empowering pharmacists to contribute more effectively to PJDs management (Bosson et al. 2023; Cottrell et al. 2021; Kredo et al. 2020). This approach supports the overarching healthcare objective of delivering patient-centred, multidisciplinary care for chronic musculoskeletal conditions (Takahashi et al. 2019). Assessing pharmacists’ understanding of the roles of other HCPs in managing musculoskeletal conditions is essential for identifying opportunities to enhance interprofessional collaboration (Mujtaba & Gazerani 2024; Taylor et al. 2022). Given the limited local evidence, this study was intended to assess South African pharmacists’ current practices and referral behaviour in PJD management. The secondary objective was to evaluate the pharmacists’ knowledge of the contributions of other HCPs involved in PJD management and their collaborative readiness. The findings may inform the development of practical and evidence-based referral guidelines.

## Research methods and design

### Study design

A cross-sectional survey design was utilised to investigate community pharmacists’ current practices and knowledge regarding the management of PJDs. The study also assessed referral practices and pharmacists’ familiarity with the roles of other HCPs in PJD management.

### Study setting and population

The research was conducted among community pharmacists practising across all nine provinces in South Africa, ensuring geographic representation and aiming to reflect the diversity of community pharmacy practice nationally. Pharmacists working in other pharmacy sectors, such as hospital, wholesale or manufacturing, were excluded unless they worked as locums in community pharmacies. Although hospital pharmacists dispense medication and engage directly with patients, they were excluded because their work is confined to hospital settings, where they collaborate with healthcare teams in a controlled environment to optimise medication use, ensure safe medicine administration and provide clinical patient care; they also interact with patients who have already consulted either a medical doctor or a nurse (Amedi & Gazerani 2024; Mohiuddin 2019). Lastly, pharmacy support personnel, comprising pharmacists’ assistants (Basic and Post-Basic) and pharmacy technicians, were excluded despite their roles in interacting with patients and dispensing medication in community pharmacies (Amedi & Gazerani 2024). According to the South African Pharmacy Council (SAPC) Scope of Practice and the *Pharmacy Act 53 of 1974*, these cadres may dispense medicines and provide basic patient counselling only under the direct supervision of a registered pharmacist (SAPC 2024); they do not have the authorisation to perform clinical tasks or provide independent patient counselling (Amedi & Gazerani 2024). Data collection commenced on 22 August 2023 and concluded on 29 September 2024, providing participants with sufficient time to complete their responses.

### Sample size and sampling technique

The sample size was calculated based on the estimated total number of community pharmacies in South Africa, which were approximately 2500 as at November 2021, according to the SAPC’s pharmacies by sector statistics. The number of community pharmacies was used because it is a legal requirement for each pharmacy to have at least one pharmacist present (Balraj et al. 2024). The number of registered pharmacists was not used because the Bachelor of Pharmacy degree in South Africa is a generalist qualification, which allows pharmacists to move across various sectors of pharmacy. Further, the SAPC database or register might not be up-to-date with the number of pharmacists working in a particular sector at any point in time (Gray, Riddin & Jugathpal 2016). A margin of error of 2.5% and a 95% confidence level were used; a target sample size (*n*) = 952 community pharmacists was determined, with an expected response of 286, which is 30% of the sample size. Purposive sampling was employed.

### Data collection instrument

A structured questionnaire was developed using the REDCap^®^ version 14.8.3 web-based system hosted at the University of the Witwatersrand to gather data via closed- and open-ended questions. Prior to completing the questionnaire, participants had to go through a study information sheet (Online Appendix 1) and provide informed consent electronically by signing the participant consent sheet (Online Appendix 2). Both documents were hosted on the secure REDCap^®^ online platform and made accessible electronically. The questionnaire included closed-ended questions to assess demographic details, current PJD management practices, referral behaviours and knowledge of the roles of other HCPs (Online Appendix 3). Open-ended questions were included to capture additional insights and participant recommendations.

The questionnaire was pretested through a pilot study involving seven community pharmacists to validate the questionnaire’s clarity and relevance. Feedback from the pilot informed refinements to question wording, sequencing and response options to enhance comprehensibility and contextual appropriateness. Internal consistency reliability was examined using preliminary Cronbach’s alpha values, with coefficients above 0.70 considered acceptable (Menon et al. 2021). The final instrument was therefore deemed valid and reliable for use in the main study. The responses from the pilot study were excluded from the main study.

### Variables measured

The key variables included were:

- Demographics: age, gender, years in practice, province and university educational background.
- Knowledge: familiarity with PJDs and the role of other HCPs.
- Practices: use of management guidelines, pharmacological and non-pharmacological recommendations and referral practices.
- Barriers: challenges in interprofessional collaboration and referral processes.

### Data collection procedure and recruitment

The questionnaire was administered electronically using an online survey platform and distributed through professional associations, WhatsApp^®^ groups identified through professional affiliations and open groups such as those for pharmacy locums, emails to pharmacies searched online, physically visiting some of the pharmacies and social media channels.

### Data analysis

The quantitative data were analysed using STATA^®^ SE 18 statistical software. Quantitative data were analysed using descriptive statistics, such as frequencies and percentages, to summarise participant characteristics and responses. The REDCap^®^ version 14.8.3 web-based system was used to build and manage the online survey; the data were extracted from the system in Microsoft^®^ Excel^®^ version 2411 comma-separated values format. The open-ended responses were analysed using content analysis to identify key trends and patterns, supported by the use of MAXQDA software 24.9.

### Ethical considerations

Ethical approval was obtained from the University of Witwatersrand Human Research Ethics Committee (Medical) with clearance certificate number: M220344. Participants provided informed consent electronically, where they were able to review the information sheet, indicate their consent to participate, enter the date of consent and append their signature using a touchpad or mouse. Confidentiality was maintained by anonymising responses, and all data were stored securely.

## Results

There were 451 participants who attempted the survey, and 373 (82.7%) completed the survey (socio-demographics summarised in Online Appendix 4). Of the 373 participants, 227 (60.9%) were female. The racial profile indicated that 204 (54.7%) were black (African people), followed by 99 (26.5%) who were white people. The age group of the participants showed that 177 (47.5%) were 29 years and under, while 28 (7.5%) of the participants were aged 60 years and above. The full demographic breakdown is provided in Online Appendix 4.

As noted in Online Appendix 4, 130 (34.8%) of the participants were practising in the Gauteng province, which is a densely populated urbanised province, while there were nine (2.4%) in the Northern Cape province, which is a rural province that is sparsely populated. The participants were asked to indicate how long they had been working in a community pharmacy, and 144 (38.6%) indicated that they had been working in a community pharmacy for under 2 years, while 36 (9.7%) had been working in a community pharmacy for over 30 years. There were 74 (19.8%) participants who obtained their undergraduate pharmacy qualification from North-West University, which was the highest, and only 17 (4.6%) from Tshwane University of Technology, which was the lowest; the rest of the data are captured in Online Appendix 4.

In addition, the participants indicated the year in which they completed their undergraduate qualification, ranging from 1974 to 2023, with 137 (36.7%) graduating during the 2010–2019 period. Furthermore, 272 participants (72.9%) reported not having a postgraduate qualification. These results are included in Online Appendix 4. Those with post-graduate qualifications indicated qualifications such as Primary Care Drug Therapy (PCDT), Diploma in Pharmacy Management, Master’s in public health, radiopharmacy, clinical pharmacy, pharmacy administration and policy regulation, business administration and PhD.

The participants were provided with the definition of PJDs and asked if they are familiar with these conditions. There were 315 (84.5%) participants who stated that they were familiar with these conditions, 49 (13.1%) stated that they were not familiar, while nine (2.4%) were somewhat familiar. Online Appendix 4 indicates the responses to the use of a guideline for managing PJD, with 229 (61.4%) stating ‘No’ and 144 (38.6%) responding ‘Yes’, specifying the use of the Department of Health’s Standard Treatment Guidelines (STGs), South African Medicine Formulary (SAMF), EMGuidance, Medscape, Monthly Index of Medical Specialities (MIMS), World Health Organization (WHO) Analgesic Ladder, journals and National Institute for Health and Care Excellence (NICE) UK guidelines. The Chi-square test of independence was used to test the null hypothesis (H_0_): There is no association between using guidelines and familiarity with the roles of other HCPs. The Chi-Square statistic was 0.0022, with a *p*-value of 0.9629. The significance level was 0.05. The odds ratio (OR) was 1.017 (95% confidence interval: 0.63–1.63), with a relative risk (RR) of 1.002 (95% confidence interval: 0.95–1.06).

No significant association was found between graduation timing (pre-2013 vs. post-2013) and familiarity with PJDs, as the Chi-square statistic was 0.4198, with a *p*-value of 0.8107. Furthermore, there was no significant association between having a postgraduate qualification and the level of familiarity with PJD. The Chi-square statistic was 1.4574, with a *p*-value of 0.4825.

**FIGURE 1:**
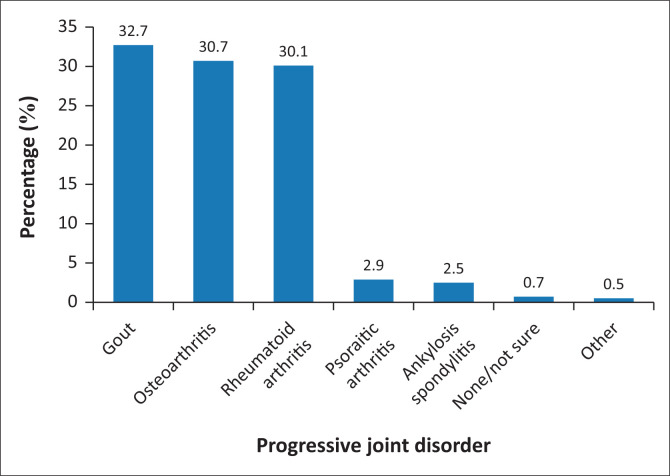
Progressive joint disorders encountered the most (*n* = 1010).

The participants reported on the PJD that they encountered the most in the pharmacies based on their familiarity with the conditions, with each participant allowed to select more than one option, as applicable (*n* = 1010). Figure 1 indicates that the three most common PJDs were gout (330; 32.7%), osteoarthritis (310; 30.7%) and rheumatoid arthritis (304; 30.1%). There were five participants (0.5%) who selected ‘Other’, where back pain, lupus erythematosus and sports injuries were specified.

**FIGURE 2:**
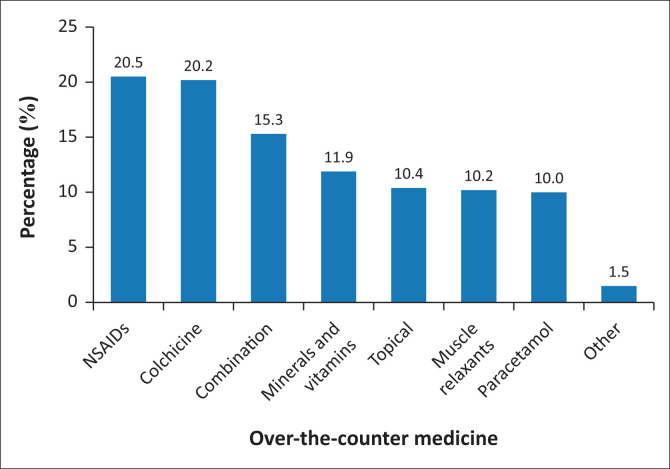
The most frequently recommended over-the-counter medicine for patients (*n* = 1423).

The participants reported on the over-the-counter medicine(s) that they commonly recommend for patients with PJD, with each participant allowed to select more than one option, as applicable, as illustrated in Figure 2 (*n* = 1423). It was reported that non-selective, non-steroidal anti-inflammatory drugs (NSAIDs) were selected by 291 (20.5%) participants, colchicine was selected by 288 (20.2%) participants, while combination products, which may contain NSAIDs/opioids/paracetamol/muscle relaxants, were selected by 218 (15.3%) participants. Mineral and vitamins supplementation were selected by 169 (11.9%) participants. The participants had an option to select ‘other’ medicine, whereby 22 (1.5%) listed other medicines such as Piascledine (5), Posteon (3), Flexofend (3), alkaloids (2) and xanthine oxidase inhibitors (9).

The participants were asked, in an open-ended question, what their next step would be should they notice that a particular patient repeatedly purchases a certain item for PJDs, and the majority opted to refer the patient to a doctor (223; 59.7%), while 50 (13.4%) chose to counsel, investigate and advise the patient without recommending medication. Some participants indicated that they would counsel and refer the patient to an appropriate HCP, which they deem fit for the management of the PJD 42 (11.3%), and others reported that they would counsel, investigate, advise and recommend medication 34 (9.1%). A smaller proportion stated they would counsel, provide alternative medication and refer to an appropriate HCP 20 (5.4%). A few participants four (1.1%) did not provide a meaningful answer, entering only a character (e.g. a full stop) to bypass the compulsory text field.

The participants reported on the counselling advise they provide to patients with PJDs, and the responses were categorised according to the themes that emerged, where 241 (23.9%) reported that they counsel patients on proper or balanced diet (including avoidance of certain food/substances), 206 (20.4%) would advise the patients on mild-to-moderate exercises and 169 (16.8%) reported that they would counsel patients on their medication (including dependence). Recommendation on the use of complementary and alternative medicine was stated by 83 (8.2%) of the participants. Sixty-eight (6.7%) participants provide detailed information of the conditions, 66 (6.5%) usually refer the patients to the doctor, while 61 (6.1%) review and dispense orthodox medicine. Advising the patient to lose weight was stated by 48 (4.8%) participants. The use of assistive devices, heat/cold therapy and other physical measures was stated by 44 (4.4%) of the participants. Lastly, there were 21 (2.1%) participants who stated that they would advise patients to consult other HCPs such as physiotherapists, dieticians, biokineticists, etc.

**FIGURE 3:**
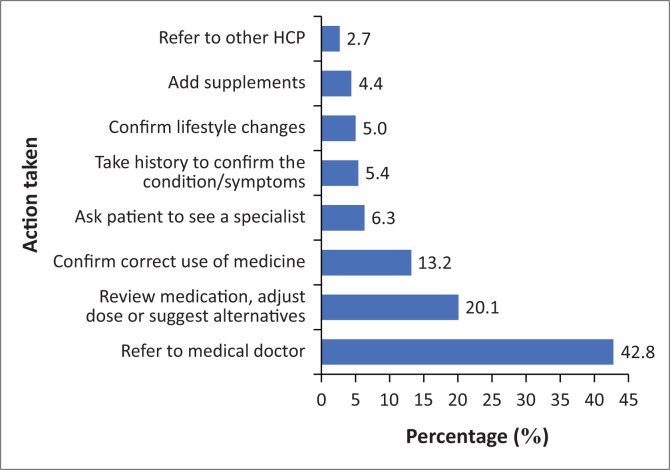
Action taken once the patient reports that the medicine taken is not assisting (*n* = 521).

The participants also provided responses regarding their actions or next steps if a patient reported that their current medication was not effective. The responses were categorised into emerging themes, with participants indicating that they would take one or more of the steps outlined in Figure 3 (*n* = 521). Referral to a medical doctor was the most common response, selected by 223 participants (42.8%), followed by reviewing the medication, adjusting the dose or suggesting alternatives, chosen by 105 participants (20.1%). Only 14 participants (2.7%) indicated that they would refer the patient to other HCPs, such as physiotherapists, dieticians, biokineticists, chiropractors, etc.

**FIGURE 4:**
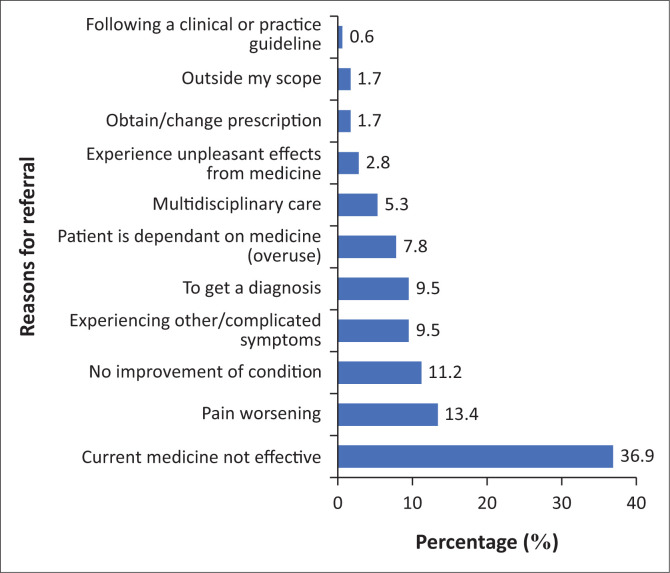
Reasons for referral (*n* = 358).

The participants were asked for the reasons for referring patients. This was an open-ended question where the responses were categorised according to the emerging themes and summarised in Figure 4 (*n* = 358). The participants indicated that they refer patients because of the medicine not being effective (132; 36.9%), the pain is worsening (48; 13.4%) and no improvement of the condition (40; 11.2%). Only two (0.6%) participants reported referring patients while following clinical or practice guidelines.

Participants reported their frequency of patient referrals to various HCPs across six categories: ‘at least weekly’, ‘at least monthly’, ‘a few times a year’, ‘never but would consider’, ‘never’ and ‘uncertain’. A total of 177 participants (47.4%) indicated that they referred patients to medical doctors on a weekly basis, while the lowest weekly referrals were reported for occupational therapists, acupuncturists and osteopaths, with only four (1.1%) participants in each group. A Chi-square test showed a significant association between provider type and referral frequency (χ^2^ = 1485.85, degrees of freedom = 35, *p* < 0.001; Cramer’s V = 0.32), indicating non-uniform referral patterns. Medical doctors were referred to significantly more often than any other HCP with occasional referrals to physiotherapists and infrequent or uncertain referrals to other HCPs such as acupuncturists, osteopaths and podiatrists. The results are summarised in Table 1.

**TABLE 1:** Pharmacist’s referral frequencies to various healthcare providers.

Frequency/healthcare provider	Weekly		Monthly		A few times a year		Never but would consider		Never		Uncertain	
	*n*	%	*n*	%	*n*	%	*n*	%	*n*	%	*n*	%
Physiotherapists	35	9.4	53	14.2	123	33.0	74	19.8	71	19.0	17	4.6
Occupational therapists	4	1.1	24	6.4	60	16.1	**113**	**30.3**	141	37.8	31	8.3
Acupuncturists	4	1.1	4	1.1	40	10.7	107	28.7	**174**	**46.6**	44	11.8
Chiropractors	7	1.9	21	5.6	78	20.9	86	23.1	145	38.9	36	9.6
Osteopaths	4	1.1	21	5.6	42	11.3	105	28.1	145	38.9	56	15.0
Biokineticists	5	1.3	25	6.7	56	15.0	**114**	**30.6**	128	34.3	45	12.1
Podiatrists	6	1.6	23	6.2	75	20.1	102	27.3	131	35.1	36	9.6
Medical doctors	**177**	**47.4**	117	31.4	63	16.9	5	1.3	6	1.6	5	1.3

Note: Bold = notably high.

The participants indicated their familiarity with the role of other HCPs (excluding medical doctors) in the management of PJD, as well as whether they refer or recommend patients to these providers. A total of 232 (62.2%) participants reported being familiar with the role of some HCPs, while 107 (28.7%) stated they are familiar with the role of most HCPs. Additionally, 32 (8.6%) indicated that they do not know the role of other HCPs, and two (0.5%) reported that they never refer patients to these HCPs.

The participants were asked to indicate which musculoskeletal conditions they would refer to various HCPs (Table 2). The most frequently referred conditions were osteoarthritis (*n* = 215), back pain (which includes neck pain, slipped discs, scoliosis, sciatica and other spinal issues) (*n* = 156) and rheumatoid arthritis (*n* = 156). The participants were uncertain about the conditions appropriate for referral to osteopaths, podiatrists and acupuncturists, with 246, 182 and 176 participants choosing this option, respectively. There were 153 participants who stated that they refer all musculoskeletal conditions to medical doctors. A Chi-square test revealed a significant association between condition type and referral destination (χ^2^ = 2456.15, degrees of freedom = 98, *p* < 0.001; Cramer’s V = 0.38), confirming that referral patterns differed significantly across provider categories.

**TABLE 2:** Musculoskeletal conditions referred to various healthcare providers.

Condition/healthcare provider	Physiotherapists		Occupational therapists		Acupuncturists		Chiropractors		Osteopaths		Biokineticists		Podiatrists		Medical doctors		Total
	*n*	%	*n*	%	*n*	%	*n*	%	*n*	%	*n*	%	*n*	%	*n*	%	
Gout	9	7.6	12	10.1	6	5.0	6	5.0	4	3.4	6	5.0	36	30.3	40	33.6	119
Osteoarthritis	61	28.4	25	11.6	19	8.8	21	9.8	24	11.2	29	13.5	10	4.7	26	12.1	215
Rheumatoid arthritis	38	24.4	42	26.9	10	6.4	16	10.3	0	0.0	18	11.5	9	5.8	23	14.7	156
Psoriatic arthritis	4	40.0	3	30.0	0	0.0	2	20.0	0	0.0	0	0.0	0	0.0	1	10.0	10
Ankylosing spondylitis	29	50.9	8	14.0	8	14.0	10	17.5	2	3.5	0	0.0	0	0.0	0	0.0	57
Muscle pain, injuries, spasms, stiffness and other complaints	50	34.5	14	9.7	14	9.7	23	15.9	5	3.4	24	16.6	0	0.0	15	10.3	145
Neck and back pain, slipped disk, scoliosis, sciatica and other spinal conditions	25	16.0	11	7.1	12	7.7	98	62.8	4	2.6	6	3.8	0	0.0	0	0.0	156
Rehabilitation/postoperative recovery	23	29.1	45	57.0	3	3.8	0	0.0	0	0.0	0	0.0	0	0.0	8	10.1	79
Trauma-related (sports injuries, sprains and strains)	18	18.8	8	8.3	0	0.0	8	8.3	10	10.4	37	38.5	5	5.2	10	10.4	96
Osteoporosis	4	28.6	10	71.4	0	0.0	0	0.0	0	0.0	0	0.0	0	0.0	0	0.0	14
Carpal tunnel syndrome, wrist and hand joint complaints	0	0.0	12	44.4	0	0.0	0	0.0	10	37.0	5	18.5	0	0.0	0	0.0	27
Foot conditions (plantar fasciitis, bunions, nail problems and diabetic foot)	0	0.0	0	0.0	0	0.0	0	0.0	0	0.0	0	0.0	25	100	0	0.0	25
All musculoskeletal conditions	8	4.4	3	1.7	2	1.1	3	1.7	0	0.0	10	5.6	1	0.6	153	85.0	180
Not sure	83	8.3	98	9.8	176	17.6	104	10.4	246	24.6	77	7.7	182	18.2	36	3.6	1002
None	0	0.0	0	0.0	17	15.3	21	18.9	14	12.6	21	18.9	33	29.7	5	4.5	111

**FIGURE 5:**
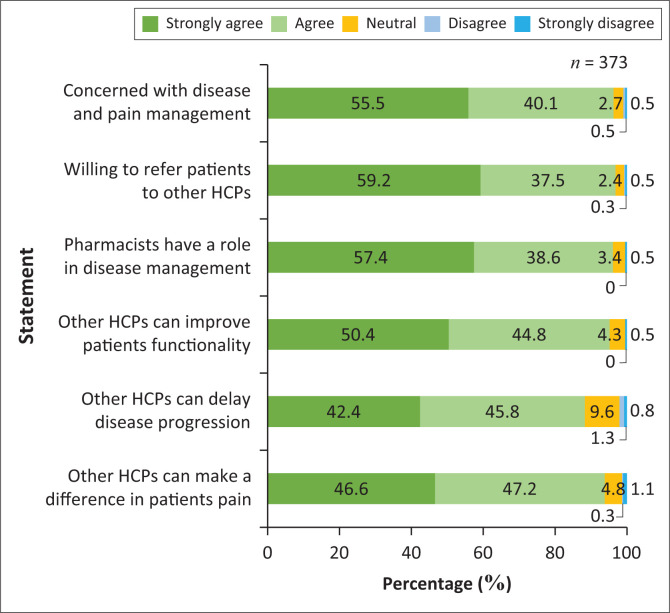
Healthcare provider’s role in managing progressive joint disorders.

Figure 5 presents the distribution of respondents’ agreement levels on several statements related to the role of HCPs in managing PJDs. The first statement was to determine whether HCPs can make a difference in the patient’s pain management; 174 (46.6%) participants strongly agreed, while 176 (47.2%) participants agreed. For the statement on HCPs making a difference in the progression of PJDs, there were 158 (42.4%) participants who strongly agreed, and 171 (45.8%) participants who agreed. Regarding the impact of HCPs on the functionality (daily activities/mobility) of patients with PJDs, none of the participants disagreed with this statement. However, two (0.5%) participants strongly disagreed. There were 214 (57.4%) participants who strongly agreed that pharmacists have a role to play in the management of these conditions. Similarly, 207 (55.5%) participants strongly agreed that they are concerned about disease and pain management in patients living with PJDs.

The participants were asked if any of their patients mentioned attending any other HCP, excluding medical doctors, for the management of their PJD. A total of 249 participants (66.7%) responded with ‘Yes’, 114 (30.6%) indicated patients had not done so, while 10 (2.7%) indicated that they had not engaged in such discussions with their patients. In response to whether participants felt the need to do more for patients with PJDs, 302 participants (81.0%) answered affirmatively, while 71 (19.0%) did not see the need to do more. Following the participants selection of either ‘Yes’ or ‘No’, participants were asked to provide reasons for their selection.

Proposals of what pharmacists would like to do for patients with progressive musculoskeletal conditions:

- Provided education and raised awareness about disease management (57; 15.3%).
- Proposed improved referral practices and collaboration with other HCPs (30; 8.0%).
- Suggested enhanced pain management strategies, including non-pharmacological interventions (30; 8.0%).
- Recommended lifestyle changes to improve patients’ quality of life and physical functionality (25; 6.7%).
- Advocated for better access to specialists such as rheumatologists and orthopaedic surgeons (20; 5.4%).

Reasons why pharmacists did not see the need to do more for these patients:

- Believed they were unable to slow disease progression (20; 5.4%).
- Considered it outside their professional scope or felt underqualified (10; 2.7%).
- Perceived the patients’ condition or pain was already under control, with no further intervention needed (9; 2.4%).
- Reported that counselling was not appreciated by patients (3; 0.8%).
- Expressed uncertainty about what more could be done (20; 5.4%).
- Did not provide any specific reason for their perspective (140; 37.5%).

## Discussion

The study assessed South African community pharmacists’ current practices in the management of PJDs, specifically examining their familiarity with these conditions, the use of guidelines, pharmacological and non-pharmacological recommendations and their referral practices to other HCPs.

### Participant characteristics

In our survey, the majority of participants who completed the study were female (60.9%), which aligns with SAPC statistics indicating that as of May 2025, approximately 66.3% of registered pharmacists in South Africa were female (SAPC 2025). The racial composition of participants (54.7% black/African people and 26.5% white people, with the remainder of Indian, Asian and coloured backgrounds) reflects South Africa’s diverse population. This distribution varies from the overall SAPC register, where the majority of pharmacists are white people (40.2%), followed by black/African people (34.6%) (SAPC 2025). The underrepresentation of white pharmacists in our survey might be because of response bias; it is possible that some demographic groups were more willing or available to participate, or it could simply reflect chance. The age distribution of our respondents was skewed towards younger pharmacists, with 47.5% being 29 years or younger, and nearly 45% having less than 2 years of work experience. This likely results from our survey dissemination methods (heavy reliance on digital platforms such as email, WhatsApp and social media), which younger pharmacists may be more engaged with, as well as the possibility that early-career pharmacists were more interested in the topic or had time to respond (Bosson et al. 2023; Mohiuddin 2019).

Further analysis revealed a significant disparity in age distribution across racial groups. Among white pharmacists, 43 out of 99 (43.4%) were over the age of 50, whereas only 28 of 274 non-white pharmacists (10.2%) were above 50. A Chi-square test confirmed a strong association between race and age distribution (χ^2^ = 49.93, *p* < 0.001). This skew likely reflects broader historical and systemic influences in South Africa. During the apartheid era, access to pharmacy education (and higher education in general) was disproportionately limited for non-white South Africans, resulting in an older cohort of pharmacists that is predominantly white people. Since the end of apartheid in 1994, transformation policies have expanded access to education for black, coloured and Indian individuals, leading to a newer generation of younger pharmacists from these groups entering the profession (DHET 2020; Mosiane et al. 2022).

### Familiarity with progressive joint diseases and commonly encountered conditions

The survey found that 84.5% of pharmacists were familiar with PJDs. Graduation timing (whether before or after 2013) and the possession of a postgraduate qualification do not appear to influence a pharmacist’s familiarity with PJDs. The distinction between pre- and post-2013 graduates was made because, in 2013, the Bachelor of Pharmacy curriculum in South Africa was formally aligned with the Higher Education Qualifications Sub-Framework (HEQSF), introducing a national shift towards outcome-based education (Mosiane et al. 2022; SAPC 2021). The observed differences in familiarity levels are likely because of random variation rather than any systematic effect of graduation timing or postgraduate education. This does not mean that postgraduate education is not beneficial. A study examining changes in pharmacists’ professional activities after completing a postgraduate qualification revealed that most participants believed their acquired skills were effectively applied in practice (Aston & Black 2009). The study further highlighted that postgraduate education improved confidence, evidence-based practice, reflective practice and critical thinking as essential and beneficial for their professional development (Aston & Black 2009). However, it has been demonstrated that the Bachelor of Pharmacy degree in South Africa equips graduates with the necessary knowledge to manage PJD, as these conditions are comprehensively covered by all the universities offering the Bachelor of Pharmacy degree (Modau, Constantinou & Orchard 2025). This is consistent with studies conducted in Canada, where pharmacists are frequently involved in screening and recommending treatments for joint conditions, including over-the-counter analgesics, and have been shown to improve patient outcomes through appropriate management and follow-up (Robinson 2019).

However, research also highlights that gaps exist in pharmacists’ knowledge, particularly regarding non-pharmacological interventions and the management of more complex cases, such as inflammatory arthritis (Cottrell et al. 2021; Robinson 2019). While pharmacists are generally familiar with first-line treatments for conditions like osteoarthritis, they may lack the specialised knowledge required for comprehensive management or for effectively referring patients to other specialists, such as physiotherapists (Cottrell et al. 2021; Robinson 2019). Furthermore, although pharmacists are capable of addressing the physical aspects of joint pain, such as recommending medications and lifestyle changes, their ability to manage these conditions holistically is often hindered by a lack of formal training and support (Cottrell et al. 2021; Robinson 2019). It is important to note that this study does not imply that pharmacists are expected to diagnose PJDs. Rather, their role lies in supporting the ongoing management of patients who have already been diagnosed and in signposting individuals with potential red flag symptoms for further medical evaluation.

The most commonly encountered PJD was gout, reported by 32.7% of the pharmacists, followed by osteoarthritis, 30.7% and rheumatoid arthritis, 30.1%. This aligns with a study conducted in the United Kingdom, which identified gout as the most common form of inflammatory arthritis, with rising prevalence rates, often managed in community pharmacies because of its recurring acute flares and chronic complications (Dickson 2022). Similarly, osteoarthritis and rheumatoid arthritis are known to be among the most prevalent PJDs globally, with osteoarthritis affecting large numbers because of ageing populations and mechanical joint stress, while rheumatoid arthritis remains common because of its autoimmune nature (Alzahrani et al. 2022; WHO 2022). Furthermore, a study conducted across nine South African universities to review the curricula on musculoskeletal conditions reported that gout, osteoarthritis and rheumatoid arthritis were comprehensively covered by all universities offering the Bachelor of Pharmacy qualification (Modau et al. 2025). The comprehensive coverage of gout, osteoarthritis and rheumatoid arthritis in pharmacy curricula equips pharmacists with a strong understanding of these conditions, making them more familiar and confident in recognising them. This familiarity likely influenced pharmacists’ responses in this study, as they are more inclined to identify and report conditions they encounter frequently in practice and feel knowledgeable about.

### Pharmacists’ referral practices

The study reveals that pharmacists primarily refer patients with musculoskeletal conditions, such as osteoarthritis, back pain and rheumatoid arthritis, to physiotherapists, occupational therapists and chiropractors, indicating an awareness of the need for multidisciplinary care. However, uncertainty exists regarding referrals to osteopaths, podiatrists and acupuncturists, with many pharmacists unsure of the appropriate conditions for which to refer patients to these providers. Some providers do not recognise acupuncture and alternative therapies as valid options, hindering referrals (McKay et al. 2021). Pharmacists in this study reported a strong preference for referring patients with musculoskeletal conditions to medical doctors, with 41.0% indicating they refer all such cases to them. This trend was further supported by referral frequency data, which showed that medical doctors were the most commonly referred to providers, often on a weekly or monthly basis. In contrast, referrals to physiotherapists were reported only a few times a year by many pharmacists, and referrals to other HCPs, such as occupational therapists, chiropractors, osteopaths, biokineticists, acupuncturists and podiatrists, were even less frequent or absent altogether. Furthermore, almost 40% of pharmacists reported referring fewer than 10 patients to other HCPs over 12 months. This pattern suggests an over-reliance on doctors, possibly because of limited exposure, guidance or healthcare norms.

Only a small proportion of pharmacists (2.1%) referred patients to other HCPs such as physiotherapists, biokineticists and chiropractors, highlighting a gap in interprofessional collaboration and the underutilisation of non-pharmacological expertise in managing PJDs. Timely referrals are vital when symptoms persist, worsen or require care beyond a pharmacist’s scope, helping to prevent disease progression and reduce inappropriate analgesic use (McKay et al. 2021). Other HCPs provide critical interventions, such as exercise therapy, pain management and lifestyle modification, which improve outcomes and reduce reliance on medication (McKay et al. 2021; Mujtaba & Gazerani 2024). Yet, evidence suggests that many pharmacists still overlook their role, despite growing support for multidisciplinary approaches (Mujtaba & Gazerani 2024; Thapa et al. 2021).

Pharmacists in this study primarily referred patients because of ineffective medications (36.9%) or worsening pain (13.4%), affirming their role as first-line responders in chronic pain management (Nsengimana et al. 2022; Thapa et al. 2021). Other reasons included lack of improvement, complex symptoms and concerns over medication dependency, reflecting pharmacists’ involvement in ongoing assessment and safe medication use. Notably, only 0.6% reported using formal guidelines when referring, highlighting an opportunity to promote disease management protocols and structured referral pathways (Nsengimana et al. 2022).

Two-thirds (66.7%) of pharmacists indicated their patients had consulted other HCPs for PJD management, consistent with evidence showing patients often seek care from multiple providers simultaneously (Takahashi et al. 2019; Taylor et al. 2022). However, these efforts are often uncoordinated, risking fragmented care (Takahashi et al. 2019). Encouragingly, over 80% of pharmacists expressed a desire to offer greater support, aligning with prior research that has shown knowledge gaps despite pharmacists’ willingness to manage musculoskeletal pain (Dabbous et al. 2020).

Pharmacists who wished to do more cited goals such as improving patient education, raising awareness and enhancing interprofessional collaboration. Yet, over a third (37.5%) gave no specific reason, possibly indicating uncertainty around role boundaries in musculoskeletal care. This ambivalence may stem from high workload demands or unclear expectations in expanding responsibilities (Abdul Razzak et al. 2024).

The findings reveal strong support among pharmacists for interdisciplinary care in managing PJDs. Over 90% acknowledged the importance of other HCPs in pain management, and more than 80% agreed they can positively influence disease progression, echoing evidence that multidisciplinary care can improve outcomes and slow joint degeneration. Nearly all pharmacists recognised the role of other HCPs in enhancing patient functionality, with only 0.5% in strong disagreement. These results highlight pharmacists’ awareness of the value of collaboration with other HCPs to improve quality of life for PJD patients (Mujtaba & Gazerani 2024; Thapa et al. 2021).

### Use of guidelines

According to our study, 61.4% of pharmacists reported using some form of guideline when managing PJDs. However, chi-square, OR and RR analyses revealed no significant association between guideline use and familiarity with the roles of other HCPs. This may be because of the nature of the guidelines used, such as the STGs, SAMF, EMGuidance, MIMS and the WHO Analgesic Ladder, which primarily focus on pharmacological treatment without explicitly outlining interdisciplinary roles (Kredo et al. 2020).

The absence of multidisciplinary content in these resources likely limits their impact on referral behaviour. This highlights the need for structured, evidence-based referral guidelines that incorporate both pharmacological and non-pharmacological management strategies. Such tools could streamline care, support early referral, reduce healthcare costs and improve collaboration across disciplines (Cottrell et al. 2021; Dixon et al. 2023).

Additionally, keeping up with new evidence remains a challenge for practitioners, which can lead to potential gaps in optimal care delivery (Dixon et al. 2023). Tailored referral protocols could enhance consistency, clarify pharmacists’ roles in musculoskeletal care and alleviate pressure on public healthcare services through more effective community-level triage (Makhavhu, Masala-Chokwe & Ramukumba 2024). Despite these advantages, no such referral guidelines currently exist in South Africa for pharmacist-led management of PJD, and no studies have explored their specific use in this context.

### Pharmacological, non-pharmacological recommendations and counselling approaches

The majority of pharmacists (78.2%) recommended non-selective NSAIDs as the primary over-the-counter treatment for PJDs, followed closely by colchicine (77.2%) and combinations of NSAIDs with paracetamol, opioids or muscle relaxants (58.5%). Non-steroidal anti-inflammatory drugs remain a preferred first-line option for PJDs because of their analgesic and anti-inflammatory properties (NICE 2020). Colchicine, widely used for the treatment of gout and osteoarthritis, offers an affordable and effective alternative (Li et al. 2021). Combining NSAIDs with central analgesics can enhance pain control while minimising adverse effects (Cottrell et al. 2021; Simons et al. 2022). However, concurrent use with corticosteroids is discouraged because of the heightened risk of gastrointestinal complications (Cottrell et al. 2021; Simons et al. 2022).

Over 45% of pharmacists recommended mineral and vitamin supplements, particularly those containing vitamins D and K and calcium, reflecting their emerging role in musculoskeletal health (Birlik 2023; WHO 2022). While strong evidence supports the use of vitamin D and calcium in managing bone health and osteoarthritis, the effectiveness of other supplements remains inconclusive (Aaseth et al. 2024; Lips et al. 2010). Therefore, supplementation should be patient specific and guided by current evidence in collaboration with other HCPs (Bigham 2023).

Pharmacists employed diverse counselling strategies. Dietary advice was the most common approach (23.9%), particularly for gout management, emphasising the avoidance of purine-rich and inflammatory foods in favour of whole, nutrient-rich diets (Tsigalou et al. 2020). Exercise counselling (20.4%) also featured prominently, supporting evidence of the benefits of physical activity for joint function (Alzahrani et al. 2022). Additionally, 16% of pharmacists addressed proper medication use and dependency risks, underscoring their role in preventing analgesic misuse (Mujtaba & Gazerani 2024; Thapa et al. 2021).

The study has demonstrated that pharmacists will benefit from clear referral guidelines within a structured and nationwide referral system, which will aid the evolving community care workforce and ensure that patients self-presenting at pharmacies can be formally and appropriately referred (Simons et al. 2022). The implementation of a referral pathway between other HCPs and pharmacists should be established so that medication-related issues may also be directed towards the pharmacist, reducing some of this burden for the medical doctors and reducing patients’ costs (Mujtaba & Gazerani 2024; Simons et al. 2022).

### Strengths and limitations

The study provides valuable insights into the role of pharmacists in managing PJDs, offering a comprehensive view of their recommendations, counselling and referral practices. By highlighting gaps in interprofessional referrals and the limited use of multidisciplinary care, the study identified critical areas for improvement while contributing to global discussions on evidence-based pharmacist-led interventions. However, the reliance on self-reported data introduces potential recall and social desirability biases, and the use of convenience sampling limits the generalisability of the findings. Additionally, the study does not assess clinical outcomes to evaluate the impact of pharmacists’ practices on patient care, and the underrepresentation of older pharmacists because of digital survey distribution may have skewed the results. Despite these limitations, the study serves as a foundation for addressing gaps in PJD management and improving pharmacist-initiated multidisciplinary care.

### Recommendations for future research

Future research should explore the perspectives of other HCPs to enhance interprofessional collaboration in PJD management while also investigating pharmacists’ educational needs regarding non-pharmacological and multidisciplinary approaches. Studies linking pharmacists’ interventions to patient outcomes are essential to assess their impact on disease progression and quality of life. Exploring the route followed by patients who are being treated by these HCPs will also provide valuable insights into the effectiveness of referrals. When patients with progressive joint diseases receive appropriate and timely management, clinical outcomes such as reduced pain, improved joint function, slower disease progression, enhanced mobility and better overall quality of life are more likely to be achieved. Additionally, research should address barriers to effective referrals, develop structured, locally relevant guidelines and compare South African practices with global standards to identify best practices.

## Conclusion

The study highlighted the significant role pharmacists play in managing PJD through medication recommendations, patient counselling and referrals while also identifying gaps in familiarity with certain HCPs and the use or availability of guidelines to support their decision-making. The findings highlight a clear disparity in referral practices, with medical doctors being the primary referral destination; other HCPs were less commonly involved in referral practices, suggesting potential gaps in collaboration or knowledge of their roles in managing PJDs. Pharmacists expressed a strong willingness to enhance patient care by improving referral practices, providing education on disease management and adopting better pain management strategies. Many emphasised the importance of raising awareness about the limitations of over-the-counter medications, the risks of prolonged NSAID and opioid use and the benefits of supplements, alternative therapies and lifestyle changes like diet and exercise. Overall, the findings suggest a need for a better understanding of when and how to refer patients to appropriate HCPs to better support patients with PJD. This can be achieved through the introduction of referral guidelines for practising pharmacists and educational interventions that incorporate interprofessional education into the Bachelor of Pharmacy curricula.
